# Supplementary material for: Advances in HIV Gene Therapy
Source: Int J Mol Sci. 2024 Feb 28;25(5):2771. doi: 10.3390/ijms25052771 (PMC10931721; doi:10.3390/ijms25052771)
Supplement: Supplementary file 1 [file ijms-25-02771-s001.zip › ijms-2724642-supplementary.pdf]

SUPPLEMENTARY INFORMATION: Advances in HIV Gene Therapy

Table S1: **List of Approved CAR-T and Gene Products**

Type of Cell/ Gene Therapy	DRUG NAME (Year of Approval)	VECTOR/ MODE OF ACTION	INDICATION	COMPANY. Ref.
CAR-T Cell Therapies	ABECMA idecabtagene vicleucel (2021)	B-cell maturation antigen (BCMA)-directed genetically modified autologous T cell immunotherapy	Adult patients with relapsed or refractory multiple myeloma after 4 or more prior lines of therapy including an immunomodulatory agent, a proteasome inhibitor, and an anti-CD38 monoclonal antibody.	Celgene Corporation (Bristol-Myers Squibb Company) https://www.abecma.com/ [1]
	CARVYKTI ciltacabtagene autoleucel (2022)	BCMA-directed genetically modified autologous T cell immunotherapy	Adult patients with relapsed or refractory multiple myeloma after 4 or more prior lines of therapy, including a proteasome inhibitor, an immunomodulatory agent, and an anti-CD38 monoclonal antibody	Janssen Biotech, Inc. https://www.carvykti.com/ [2]
	BREYANZI, lisocabtagene maraleucel (2021)	CD19-directed genetically modified autologous T cell immunotherapy	Adult patients with large B-cell lymphoma (LBCL), including diffuse large B-cell lymphoma (DLBCL) not otherwise specified, high-grade B-cell lymphoma, primary mediastinal large B-cell lymphoma, and follicular lymphoma grade 3B with refractory disease	Juno Therapeutics, Inc., Bristol-Myers Squibb Company https://www.breyanzi.com/ [3]
	KYMRIAH tisagenlecleucel (2017)	CD19-directed genetically modified autologous T cell immunotherapy	Adult patients with relapsed or refractory follicular lymphoma after two or more lines of therapy	Novartis Pharmaceuticals Corporation https://www.hcp.novartis.com/ products/kymriah/ [4]
	TECARTUS brexucabtagene autoleucel (2020)	CD19-directed genetically modified autologous T cell immunotherapy	Adult patients with relapsed or refractory mantle cell lymphoma (MCL) and patients with relapsed or refractory (r/r) B-cell precursor acute lymphoblastic leukemia (ALL)	Kite Pharma, Inc https://www.tecartus.com/ [5]
	YESCARTA axicabtagene ciloleucel (2017)	CD19-directed genetically modified autologous T cell immunotherapy	Adult patients with large B-cell lymphoma that is refractory to first-line chemotherapy or that relapses within 12 months of first-line chemotherapy.	Kite Pharma, Inc https://www.yescarta.com/ [6]
Autologous Cell Therapy	PROVENGE sipuleucel-T (2010)	Autologous cellular immunotherapy	For treatment of asymptomatic or minimally symptomatic metastatic castrate resistant (hormone refractory) prostate cancer.	Dendreon Corporation https://provenge.com/ [7]
Single Stranded Oligonucleotide (SSO)	DEFITELIO Defibrotide (2016)	Single stranded oligonucleotide given intravenously	Treatment of severe hepatic veno-occlusive disease, with renal or pulmonary dysfunction following hematopoietic stem cell (HSC) transplantation	Jazz Pharmaceuticals https://www.defitelio.com/ [8, 9]

Type of Cell/ Gene Therapy	DRUG NAME (Year of Approval)	VECTOR/ MODE OF ACTION	INDICATION	COMPANY. Ref.
Antisense Oligonucleotides (ASO)	EXONDYS 51 Eteplirsen (US FDA, 2016)	Antisense oligonucleotide that causes skipping of Exon 51 given by intravenous infusion	For treating patients with Duchenne muscular dystrophy (DMD)	Sarepta Therapeutics https://www.exondys51.com/ [10]
	KYNAMRO Mipomersen 2013 US FDA. Rejected by EMA	2nd generation antisense oligonucleotide administered subcutaneously	Used to treat homozygous familial hypercholesterolemia by blocking the production of apolipoprotein B, the main component of LDL cholesterol	Ionis Pharmaceuticals [11]
	SPINRAZA nusinersen (2016 US FDA)	anti-sense oligonucleotide that enables SMN2 gene to produce full length protein. Administered intrathecally	For the treatment 5q spinal muscular atrophy (SMA)	Ionis Pharmaceuticals and Cold Spring Harbor Laboratory https://www.spinraza.com/ [12, 13]
Aptamer	MACUGEN Pegaptanib sodium (US FDA 2004)	Pegylated aptamer administered in vivo (intravitreal)	For the treatment of neovascular (wet) age-related macular degeneration (AMD)	NeXstar Pharmaceuticals [14]
Plasmid DNA	NEOVASCULGEN Cambiogeneplasmid (Russia, 2011)	Plasmid DNA encoding the 165-amino-acid isoform of human vascular endothelial growth factor (pCMV - VEGF165); intraneural injection (in vivo)	For peripheral artery disease, including peripheral limb ischemia	Human Stem Cells Institute, Russia [15]
Genetically modified Herpes Simplex Type 1 (Oncolytic Virus)	IMLYGIC talimogene laherparepvec (2015)	Genetically modified oncolytic viral therapy designed to replicate within tumors and to produce the immune stimulatory protein GM-CSF	For the local treatment of unresectable cutaneous, subcutaneous, and nodal lesions in patients with melanoma recurrent after initial surgery. It causes lysis of tumors, followed by release of tumor-derived antigens, which together with virally derived GM-CSF may promote an antitumor immune response	BioVex, Inc., a subsidiary of Amgen Inc. https://www.imlygic.com/ [16]
Small Interfering RNA siRNA	ONPATTRO (Patisiran,	Double stranded siRNA delivered in lipid nanoparticles and administered intravenously	For the treatment of hereditary transthyretin-mediated amyloidosis by targeting a sequence within the transthyretin messenger RNA to decrease hepatic production of mutant and wild-type transthyretin	Alnylam Pharmaceuticals https://www.onpattro.com/ [17]
	GIVLAARI (Givosiran)	Double-stranded siRNA that causes degradation of aminolevulinate synthase-1 (ALAS1) mRNA in hepatocytes through RNA interference	Treatment of adult patients with acute hepatic porphyria. Reduced ALAS-1 mRNA results in reduced levels of neurotoxic intermediates, aminolevulinic acid (ALA) and porphobilinogen (PBG) which are responsible for attacks	Alnylam Pharmaceuticals [18]
	OXLUMO (Lumasiran)	double-stranded siRNA that reduces levels of glycolate oxidase (GO) enzyme by targeting the HAO1	for the treatment of primary hyperoxaluria type 1 (PH1). Decreased GO levels eventually lead to a reduction of urinary and plasma oxalate levels	Alnylam Pharmaceuticals https://www.oxlumo.com/ [19]

Type of Cell/ Gene Therapy	DRUG NAME (Year of Approval)	VECTOR/ MODE OF ACTION	INDICATION	COMPANY. Ref.
		mRNA in hepatocytes through RNA interference		
	LEQVIO (Inclisiran)	double-stranded siRNA that acts by inhibiting translation of liver-produced proprotein convertase, PCSK9.	For the treatment of people with clinical atherosclerotic cardiovascular disease (ASCVD) and heterozygous familial hypercholesterolemia. PCSK9 degradation results in a reduction of lysosomal degradation of LDL receptors, therefore reducing the level of circulating LDL	Novartis Pharmaceuticals [20]
Adenovirus	ADSTILADRIN (nadofaragene firadenovec-vncg)	Non-replicating adenoviral vector-based therapy for the delivery of a copy of gene encoding a human interferon alpha 2b (IFNα2b) to the bladder urothelium	For the treatment of adult patients with high-risk Bacillus Calmette-Guérin (BCG)-unresponsive non-muscle invasive bladder cancer (NMIBC) with carcinoma in situ (CIS) with or without papillary tumors	Ferring Pharaceuticals A/S [21]
Oncolytic Adenovirus	GENDICINE rAd-p53 (2003)	In vivo administration of Recombinant adenovirus (Ad) vector carrying human wt p53 gene	Treatment of head and neck squamous-cell carcinoma (HNSCC)	Shenzhen SiBiono GeneTech, approved by China FDA http://www.sibiono.com/en/productinfo.aspx [22]
	ONCORINE (China FDA, 2005)	Oncolytic adenovirus -genetically modified adenovirus named H101 (*E1B*-deletion). Administered *in vivo*	Used in conjunction with chemotherapy for the treatment of nasopharyngeal carcinoma and head and neck cancer	Shanghai Sunway Biotec, China [23]
Adeno-Associated Virus Vectors	HEMGENIX (etranacogene dezaparvovec)	Adeno-Associated Virus 5 vector-based gene therapy	For the treatment of adults with Hemophilia B (congenital Factor IX deficiency) who: Currently use Factor IX prophylaxis therapy, or have current or historical life-threatening hemorrhage, or have repeated, serious spontaneous bleeding episodes.	CSL Behring LLC [24]
	LUXTURNA voretigene neparvovec-rzyl (2017)	Adeno-Associated Virus vector-based gene therapy	For treatment of patients with confirmed biallelic *RPE65* mutation-associated retinal dystrophy. First gene product in USA targeting gene mutations	Spark Therapeutics, Inc. https://luxturna.com/ [25]
	ZOLGENSMA 2019	Adeno-Associated Virus vector-based gene therapy	For the treatment of paediatric patients less than 2 years of age with spinal muscular atrophy (SMA) with bi-allelic mutations in the survival motor neuron 1 (SMN1) gene	Novartis Gene Therapies, Inc. https://www.zolgensma.com/ [26]
	Roctavian (valoctocogene roxaparvovec-rvox)	Adeno-associated virus serotype 5 (AAV5) based gene therapy vector, that introduces a functional copy of a transgene encoding the B-domain deleted SQ form of human coagulation factor VIII	For the treatment of adults with severe hemophilia A (congenital factor VIII deficiency with factor VIII activity < 1 IU/dL) without pre-existing antibodies to adeno-associated virus serotype 5 detected by an FDA-approved	BioMarin Pharmaceutical Inc. NCT04323098

Type of Cell/ Gene Therapy	DRUG NAME (Year of Approval)	VECTOR/ MODE OF ACTION	INDICATION	COMPANY. Ref.
			test.	
	Elevidys delandistrogene moxeparvovec-rokl	Adeno-associated virus delivering a gene that codes for a shortened form of dystrophin	For the treatment of ambulatory pediatric patients aged 4 through 5 years with Duchenne muscular dystrophy (DMD)	Sarepta Therapeutics Inc [27]
Retroviral vector	REXIN-G (Philipines FDA 2007)	Retrovector bearing a cytocidal negative cyclin-G1 construct administered intravenously	For the treatment of sarcoma, osteosarcoma, pancreatic cancer	Epeius Biotechnologies [28]
	STRIMVELIS (2016)	*Ex vivo* autologous CD34+ cells transduced with retroviral vector that encodes for the human adenosine deaminase (ADA)	For patients with ADA-SCID who cannot undergo bone-marrow transplant for lack of a suitable, matched, related donor.	Orchard Therapeutics [29]
Lentiviral Vector	LIBMELDY 2007	*Ex vivo* Autologous CD34+ cells genetically modified with lentiviral vector that encodes for ARSA gene	For treating children with early onset of metachromatic leukodystrophy (MLD	Orchard Therapeutics https://www.libmeldy.eu/ [30, 31]
	SKYSONA (elivaldogene autotemcel)	*Ex vivo* Autologous CD34+ cells genetically modified with lentiviral vector Lenti-D	To slow the progression of neurologic dysfunction in boys 4-17 years of age with early, active cerebral adrenoleukodystrophy (CALD)	Bluebird Bio, Inc. [32]
	ZYNTEGLO (betibeglogene autotemcel) 2019	Autologous, lentiviral *ex vivo* hematopoietic stem cell-based gene therapy	For treatment of adult and paediatric patients with ß-thalassemia who require regular red blood cell (RBC) transfusions	Bluebird Bio Inc. https://www.zynteglo.com/ [33]

Table S2: *Ex vivo* **and** *In vivo* **HIV Cell and Gene Therapy Clinical Trials; Source: https://clinicaltrials.gov**

Interventions	NCT Number; Other ID	Cells Modified	Title	Status	Sponsor/ Collaborators	Study Designs	Study Results
			**CELL THERAPY**				
*GCSF Mobilized Allogeneic PBSC Cultured w/Cytokines; Transduced w/RV	NCT00005785; 990167| 99-DK-0167	CD34 HSPC	Stem Cell (Modified Bone Marrow) Transplantation in HIV-Infected Patients With Blood Cancer	Completed Sep 1 1999	NIDDK; NIHCC	Phase1- Primary Purpose: Treatment	No Results posted
*NeoR gene delivered via retroviral vectors. Genetically Marked Syngeneic T Lymphocytes	NCT00001353; 930110| 93-I-0110	CD4	Safety and Survival of Genetically Modified White Blood Cells in HIV-Infected Persons - A Study in Identical Twin Pairs	Completed Mar 1 2002	NIAID; NIHCC		No Results posted [175]
*Syngeneic Lymphocytes (CD4+) Cultured with OKT3 (Ortho) and Interleukin-2 (Chiro)	NCT00001535; 960051| 96-HG-0051	CD4	Twins Study of Gene Therapy for HIV Infection	Completed Mar 1 2002	NHGRI; NIHCC	Phase1- Primary Purpose: Treatment	No Results posted
WT-gag-TCR modified T cells or α/6-gag-TCR modified T cells	NCT00991224; 810108	CD8 (SL9 TCR)	Redirected High Affinity Gag-Specific Autologous T Cells for HIV Gene Therapy	Completed Jan 1 2014	University of Pennsylvania; Adaptimmune	Phase1- Interventional: Non-Randomized; Factorial Assignment; Masking: None	No Results posted
AGT103-T-LTFU	NCT05529342; AGT103-T-LTFU	CD4	Long-term Follow-up of Study Participant Treated With Lentiviral-Based Genetically Modified Autologous Cell Product, AGT103-T	Enrolling by invitation	American Gene Technologies International Inc.	Observational: Prospective, Case-Control	No Results posted
(AGT103-T): Antiretroviral Therapy Interruption (ATI)	NCT05540964; AGT-HC-169	CD4	An Antiretroviral Treatment Interruption (ATI) Study to Evaluate the Impact of Genetically Modified Autologous Cells (AGT103-T) to Suppress HIV Replication in the Absence of Antiretroviral Therapy	Enrolling by invitation	American Gene Technologies International Inc.	Observational: Case-Control, Prospective	No Results posted
AGT103-T	NCT04561258; AGT-HC168. NCT03215004.	CD4	Study to Evaluate the Safety of a Gene and Cell Therapy Product in Participants with HIV That is Well-Controlled on Antiretroviral Therapy	Recruiting	American Gene Technologies International Inc.	Phase1 -Interventional: Non-Randomized;Parallel Assignment; Masking: None	Initial results [173]

Interventions	NCT Number; Other ID	Cells Modified	Title	Status	Sponsor/ Collaborators	Study Designs	Study Results
SB-728: ZFN modified T cells	NCT00842634; 806383	CD4	Autologous T-Cells Genetically Modified at the CCR5 Gene by Zinc Finger Nucleases SB-728 for HIV	Completed Jan 13 2013	University of Pennsylvania; ST	Phase1- Interventional: Non-Randomized; Parallel Assignment; Masking: None	No Results posted
Genetic: SB-728-T	NCT01044654; SB-728-0902	CD4	Phase 1 Dose Escalation Study of Autologous T-cells Genetically Modified at the CCR5 Gene by Zinc Finger Nucleases in HIV-Infected Patients	Completed Dec 1 2014	Sangamo Therapeutics	Phase1- Interventional: Non-Randomized Single Group Assignment; Masking: None	No Results posted
SB-728-T	NCT01252641; SB-728-1002	CD4	Study of Autologous T-cells Genetically Modified at the CCR5 Gene by Zinc Finger Nucleases in HIV-Infected Subjects	Completed May 1 2015	ST	Phase 1/2- Interventional: Single Group Assignment; Masking: None	No Results posted
SB-728mR-T|Drug: Cyclophosphamide	NCT02225665; SB-728mR-1401<br>NCT02388594	CD4	Repeat Doses of SB-728mR-T After Cyclophosphamide Conditioning in HIV-Infected Subjects on HAART	Completed Jun 1 2018	ST	Phase 1/2- Interventional: Non-Randomized; Single Group Assignment; Masking: None	Has Results; [174]
SB-728mR-HSPC Infusion 3 days following busulfan conditioning	NCT02500849; 14017	CD34 HSPC	Safety Study of Zinc Finger Nuclease CCR5-modified Hematopoietic Stem/Progenitor Cells in HIV-1 Infected Patients	Active, not recruiting	City of Hope MC; ST; CIRM	Phase1- Interventional: Non-Randomized; Single Group Assignment; No Masking	No Results posted
SB-728-T: Expanded unmodified autologous CD4+ T cells	NCT03666871; TRAILBLAZER	CD4	CCR5-modified CD4+ T Cells for HIV Infection	Active, not recruiting	Univ. of Cincinnati; UCSF; CWRU	Interventional: Randomized; Parallel Assignment; Masking: Double (Participant, Investigator)	No Results posted
*SB-728-T or SB-728mR-T	NCT04201782; SB-728-1003	CD34 HSPC and CD4	Long-term Follow-up of HIV Subjects Exposed to SB-728-T or SB-728mR-T	Enrolling by invitation	ST	Observational: Cohort; Prospective	No Results posted
Autologous CD4+ T cells genetically modified with a retroviral vector expressing MazF endoribonuclease gene (MazF-T), given IV	NCT01787994; 815441	CD4	Redirected MazF-CD4 Autologous T Cells for HIV Gene Therapy	Completed Jul 1 2017	University of Pennsylvania	Phase1- Interventional: Non-Randomized; Parallel Assignment; Masking: None	No Results posted. [178]

Interventions	NCT Number; Other ID	Cells Modified	Title	Status	Sponsor/ Collaborators	Study Designs	Study Results
*CD4-ZETA Gene Modified T Cells	NCT01013415; WU #8829-99	CD4	CD4-ZETA Gene Modified T Cells With and Without Exogenous Interleukin-2 (IL-2) In HIV Patients	Completed Aug 1 2021	University of Pennsylvania	Phase1- Interventional: Non-Randomized; Parallel Assignment; Masking: None	No Results posted. [172]
Genetic: CCR5 gene modification	NCT03164135; 307-HSPC-R5	CD34 HSPC	Safety of Transplantation of CRISPR CCR5 Modified CD34+ Cells in HIV-infected Subjects With Hematological Malignances	Completed May 20 2021	Academy of Military Medical Sciences; Peking Univ.; Capital Medical Univ.	Phase, N/A - Interventional: Single Group Assignment; Masking: None	No Results posted
CD4 CAR+CCR5 ZFN T-cells	NCT03617198; 831464	CD4	CD4 CAR+ ZFN-modified T Cells in HIV Therapy	Active, not recruiting	University of Pennsylvania	Phase 1- Interventional: Randomized; Parallel Assignment; Masking: None	No Results posted
LVgp120duoCAR-T cells, low dose and high dose; Cyclophosphamide	NCT04648046; 20-31976	CD4	CAR-T Cells for HIV Infection	Recruiting	Steven Deeks; Caring Cross; UCSF	Phase 1/2-Interventional: Non-Randomized; Sequential Assignment; Masking- None	No Results posted
	NCT04799483; 020080|02-I-0080	CD4	Safety and Survival of Genetically Modified White Blood Cells in HIV-infected Twins The Gemini Study	Active, not recruiting	NIAID; NIHCC	Observational: Cohort; Prospective	No Results posted
			**GENE THERAPY**				
Retrovirus carrying multiple ribozymes	NCT00002221; STUDY 2	Peripheral blood HSC	Gene Therapy in HIV-Positive Patients With Non-Hodgkin's Lymphoma	Completed June 24 2005	Ribozyome; NIH AIDS CTIS	Phase2: Primary Purpose: Treatment	No Results posted
RevM10 gene, RevM10/polAS gene	NCT00003942; CDR0000067135|SYSTEMIX-105	CD34 HSPC	Gene Therapy, Chemotherapy, and Peripheral Stem Cell Transplantation in Treating Patients With HIV-Related Non-Hodgkin's Lymphoma	Unknown status	Systemix; NCI	Phase 1/2- Primary Purpose: Treatment	No Results posted
OZ1 transduced CD34+ cells	NCT00074997; CR010783|OZ1-HV1-201	CD34 HSPC	An Efficacy and Safety Study of Autologous CD34+ Hematopoietic Progenitor Cells Transduced With Placebo or an Anti- HIV 1 Ribozyme (OZ1) in Participants With HIV-1 Infection	Completed Jan 1 2008	Janssen-Cilag Pty Ltd	Phase2- Interventional: Randomized; Parallel Assignment; Masking: Double (Participant, Investigator)	No results posted [180]

Interventions	NCT Number; Other ID	Cells Modified	Title	Status	Sponsor/ Collaborators	Study Designs	Study Results
*Genetic: OZ1 transduced cells	NCT01177059; CR016027|OZ1-HV1-202	CD34 HSPC	Long Term Follow-Up Study of Human Immunodeficiency Virus Type 1 (HIV-1) Positive Patients Who Have Received OZ1 Gene Therapy as Part of a Clinical Trial	Completed Nov 30 2017	Janssen-Cilag Pty Ltd	Phase2- Interventional: Single Group Assignment; Masking: None	Has results
C46/CCR5/P140K Lentiviral Vector-transduced Autologous HSPCs administered with cytotoxics	NCT02343666; 2673.00|NCI-2014-02395 |2673	CD34 HSPC	HIV-Resistant Gene Modified Stem Cells and Chemotherapy in Treating Patients With Lymphoma With HIV Infection	Withdrawn	Fred Hutchinson Cancer Center; NCI; NHLBI	Phase1- Interventional: Single Group Assignment; Masking: None	No Results posted
Cal-1 modified HSPC and CD4+ T cells with Busulfan conditioning	NCT01734850; CAL-USA-11		Safety Study of a Dual Anti-HIV Gene Transfer Construct to Treat HIV-1 Infection	Completed Nov 1 2017	Calimmune, Inc.	Phase 1/2- Interventional: Non-Randomized; Parallel Assignment; Masking: None	Has results
Cal-1 (LVsh5/C46)	NCT03593187; P 141004|2015-004453-41	CD34 HSPC and CD4	A Study Evaluating the Safety of Cal-1 (LVsh5/C46) Drug Product in HIV-1 Infected Patient With High Risk Lymphoma	Completed Jul 28 2020	APHP, CSL Behring	Phase 1/2- Interventional: Single Group Assignment; Masking: None	No Results posted
*Cal-1 (LVsh5/C46)- LTFU	NCT02390297; CAL-INT-00	CD34 HSPC and CD4	Long Term Follow up for the Detection of Delayed Adverse Events in Cal-1 Recipients	Active, not recruiting	Calimmune, Inc.	Observational Model: Prospective	No Results posted
LV vector rHIV7-shI-TAR-CCR5RZ-transduced HSPC given with several cytotoxic agents	NCT00569985; 04047|CHNMC-04047|NCI-2012-00437	CD34 HSPC	Gene Therapy-Treated Stem Cells in Treating Patients Undergoing Stem Cell Transplant for Intermediate-Grade or High-Grade AIDS-Related Lymphoma	Completed Nov 12 2019	City of Hope Medical Center; NCI	Phase1- Interventional: Single Group Assignment; Masking: None	No Results posted
Busulfan; lentivirus vector rHIV7-shI-TAR-CCR5RZ-transduced HSPC	NCT01961063; 13282|NCI-2013-01728	CD34 HSPC	Gene Therapy After Frontline Chemotherapy in Treating Patients With AIDS-Related Non-Hodgkin Lymphoma	Active, not recruiting	City of Hope Medical Center	Phase1- Intervention Model: Single Group Assignment; Masking: None	No Results posted
LV Vector rHIV7-shI-TAR-CCR5RZ-transduced HSPC with Prednisone, Rituximab, and several cytotoxics	NCT02337985; 14004|NCI-2014-02416| U01CA183012	CD34 HSPC	Gene Therapy and Combination Chemotherapy in Treating Patients With AIDS-Related Non-Hodgkin Lymphoma	Active, not recruiting	City of Hope Medical Center; NCI	Phase1- Interventional: Single Group Assignment; Masking: None	No Results posted

Interventions	NCT Number; Other ID	Cells Modified	Title	Status	Sponsor/ Collaborators	Study Designs	Study Results
CRISPR/Cas9 CCR5 Gene Modification – Nucleofection	NCT03164135 307-HSPC-R5	CD34 HSPC	Safety and Feasibility Study of Allotransplantation of CRISPR/Cas9 CCR5 Gene Modified CD34+ Hematopoietic Stem/Progenitor Cells in HIV-infected Subjects With Hematological Malignances	Unknown	Affiliated Hospital to Academy of Military Medical Sciences, Beijing	Interventional: Single group Assignment	Result in 1 patient [192]
pHIV7-shI-TAR-CCR5RZ treated CD4 cells	NCT01153646; 03161 | IND #14146	CD4	Gene Transfer for HIV Using Autologous T Cells	Terminated Jan 1 2011	City of Hope Medical Center	Phase1- Interventional: Single Group Assignment; Masking: None	No Results posted
VRX496-transduced autologous CD4 T cells	NCT00622232; VRX496-USA-05-002-Rollover	CD4	A Rollover Study for Subjects Who Completed Participation in the VRX496-USA-05-002 Trial	Active, not recruiting	VIRxSYS Corporation	Phase2: Interventional: Non-Randomized; Single Group Assignment; Masking: None	No Results posted [182]
VRX496-Modified Autologous T cells (Lexgenleucel-T)	NCT00131560; VRX496-USA-05-002 NCT00295477	CD4	Safety and Efficacy of T Cell Genetic Immunotherapy for HIV	Active, not recruiting	VIRxSYS Corporation	Phase2- Interventional: Non-Randomized; Single Group Assignment; No Masking	No Results posted
LV vector CCR5 shRNA/TRIM5alpha/ TAR Decoy-transduced Autologous CD34 HSPC	NCT02797470; AMC-097 | NCI-2015-01745	CD34 HSPC	Gene Therapy in Treating Patients With Human Immunodeficiency Virus-Related Lymphoma Receiving Stem Cell Transplant	Recruiting	AIDS Malignancy Consortium; NCI; CIRM	Phase 1/2- Interventional: Single Group Assignment; Masking: None	No Results posted
shRNA-modified CD34+ cells with Low dose busulfan preconditioning	NCT03517631; KL1702	CD34 HSPC	An Efficacy and Safety Study of shRNA-modified CD34+ Cells in HIV-infected Patients.	Unknown status Dec 31 2020	SPHCC; R&D Kanglin Biotech	Phase1- Interventional: Non-Randomized; Parallel Assignment; Masking: None	No Results posted

IN-VIVO STUDIES

Interventions	NCT Number; Other ID	Cells Modified	Title	Status	Sponsor/ Collaborators	Study Designs	Study Results
Leronlimab – CCR5 Antagonist	NCT02859961 (Others - NCT00642707, NCT02175680, NCT02355184, NCT02483078, NCT02990858, NCT03902522, NCT05271370)		A Phase 2b/3, Multicenter Study to Assess the Treatment Strategy of Using PRO 140 SC as Long-Acting Single-Agent Maintenance Therapy for 48 Weeks in Virologically Suppressed Subjects With CCR5-tropic HIV-1 Infection	Unknown	CytoDyn Inc	Interventional – Treatment Open label, Randomized, Parallel Assignment	Initial results – product safe (mild side effects and efficacious at higher doses of 525 and 700mg
EBT-101 CRISPR-Cas9 delivered via AAV vector	NCT05144386		Study of EBT-101 in Aviremic HIV-1 Infected Adults on Stable ART to evaluate the safety, tolerability, biodistribution and pharmacodynamics	Recruiting	Excision BioTherapeutics	Open-label, multi-center, single ascending dose study Phase 1 – First in Human Trial	Initial results - no serious adverse events

*These studies could enrol children

Assistance Publique -Hopitaux de Paris; (APHP)
California Institute for Regenerative Medicine (CIRM)
Case Western Reserve University (CWRU)
CTIS: Clinical Trials Information Service
National Cancer Institute (NCI)
National Heart, Lung, and Blood Institute (NHLBI)
National Human Genome Research Institute (NHGRI)
National Institute of Allergy and Infectious Diseases (NIAID)
National Institute of Diabetes and Digestive and Kidney Diseases (NIDDK)
National Institutes of Health Clinical Center (NIHCC)
Sangamo Therapeutics (ST)
Shanghai Public Health Clinical Center (SPHCC);
University of California San Francisco (UCSF)

References

1. Munshi, N.C.; Anderson, L.D.; Shah, N.; Madduri, D.; Berdeja, J.; Lonial, S.; Raje, N.; Lin, Y.; Siegel, D.; Oriol, A.; et al. Idecabtagene Vicleucel in Relapsed and Refractory Multiple Myeloma. *N. Engl. J. Med.* **2021**, *384*, 705–716. https://doi.org/10.1056/NEJMoa2024850.
2. Berdeja, J.G.; Madduri, D.; Usmani, S.Z.; Jakubowiak, A.; Agha, M.; Cohen, A.D.; Stewart, A.K.; Hari, P.; Htut, M.; Lesokhin, A.; et al. Ciltacabtagene autoleucel, a B-cell maturation antigen-directed chimeric antigen receptor T-cell therapy in patients with relapsed or refractory multiple myeloma (CARTITUDE-1): A phase 1b/2 open-label study. *Lancet* **2021**, *398*, 314–324. https://doi.org/10.1016/S0140-6736(21)00933-8.
3. Abramson, J.S.; Palomba, M.L.; Gordon, L.I.; Lunning, M.A.; Wang, M.; Arnason, J.; Mehta, A.; Purev, E.; Maloney, D.G.; Andreadis, C.; et al. Lisocabtagene maraleucel for patients with relapsed or refractory large B-cell lymphomas (TRANSCEND NHL 001): A multicentre seamless design study. *Lancet* **2020**, *396*, 839–852. https://doi.org/10.1016/S0140-6736(20)31366-0.
4. Schuster, S.J.; Bishop, M.R.; Tam, C.S.; Waller, E.K.; Borchmann, P.; McGuirk, J.P.; Jäger, U.; Jaglowski, S.; Andreadis, C.; Westin, J.R.; et al. Tisagenlecleucel in Adult Relapsed or Refractory Diffuse Large B-Cell Lymphoma. *N. Engl. J. Med.* **2018**, *380*, 45–56. https://doi.org/10.1056/NEJMoa1804980.
5. Wang, M.; Munoz, J.; Goy, A.; Locke, F.L.; Jacobson, C.A.; Hill, B.T.; Timmerman, J.M.; Holmes, H.; Jaglowski, S.; Flinn, I.W.; et al. KTE-X19 CAR T-Cell Therapy in Relapsed or Refractory Mantle-Cell Lymphoma. *N. Engl. J. Med.* **2020**, *382*, 1331–1342. https://doi.org/10.1056/NEJMoa1914347.
6. Neelapu, S.S.; Locke, F.L.; Bartlett, N.L.; Lekakis, L.J.; Miklos, D.B.; Jacobson, C.A.; Braunschweig, I.; Oluwole, O.O.; Siddiqi, T.; Lin, Y.; et al. Axicabtagene Ciloleucel CAR T-Cell Therapy in Refractory Large B-Cell Lymphoma. *N. Engl. J. Med.* **2017**, *377*, 2531–2544. https://doi.org/10.1056/NEJMoa1707447.
7. Kantoff, P.W.; Higano, C.S.; Shore, N.D.; Berger, E.R.; Small, E.J.; Penson, D.F.; Redfern, C.H.; Ferrari, A.C.; Dreicer, R.; Sims, R.B.; et al. Sipuleucel-T Immunotherapy for Castration-Resistant Prostate Cancer. *N. Engl. J. Med.* **2010**, *363*, 411–422. https://doi.org/10.1056/NEJMoa1001294.
8. Richardson, P.G.; Riches, M.L.; Kernan, N.A.; Brochstein, J.A.; Mineishi, S.; Termuhlen, A.M.; Arai, S.; Grupp, S.A.; Guinan, E.C.; Martin, P.L.; et al. Phase 3 trial of defibrotide for the treatment of severe veno-occlusive disease and multi-organ failure. *Blood* **2016**, *127*, 1656–1665. https://doi.org/10.1182/blood-2015-10-676924.
9. Kernan, N.A.; Grupp, S.; Smith, A.R.; Arai, S.; Triplett, B.; Antin, J.H.; Lehmann, L.; Shore, T.; Ho, V.T.; Bunin, N.; et al. Final results from a defibrotide treatment-IND study for patients with hepatic veno-occlusive disease/sinusoidal obstruction syndrome. *Br. J. Haematol.* **2018**, *181*, 816–827. https://doi.org/10.1111/bjh.15267.
10. Lim, K.R.; Maruyama, R.; Yokota, T. Eteplirsen in the treatment of Duchenne muscular dystrophy. *Drug Des. Devel. Ther.* **2017**, *11*, 533–545. https://doi.org/10.2147/dddt.S97635.
11. Wong, E.; Goldberg, T. Mipomersen (kynamro): A novel antisense oligonucleotide inhibitor for the management of homozygous familial hypercholesterolemia. *Pharm. Ther.* **2014**, *39*, 119–122.
12. Finkel, R.S.; Chiriboga, C.A.; Vajsar, J.; Day, J.W.; Montes, J.; De Vivo, D.C.; Yamashita, M.; Rigo, F.; Hung, G.; Schneider, E.; et al. Treatment of infantile-onset spinal muscular atrophy with nusinersen: A phase 2, open-label, dose-escalation study. *Lancet* **2016**, *388*, 3017–3026. https://doi.org/10.1016/s0140-6736(16)31408-8.
13. Mercuri, E.; Darras, B.T.; Chiriboga, C.A.; Day, J.W.; Campbell, C.; Connolly, A.M.; Iannaccone, S.T.; Kirschner, J.; Kuntz, N.L.; Saito, K.; et al. Nusinersen versus Sham Control in Later-Onset Spinal Muscular Atrophy. *N. Engl. J. Med.* **2018**, *378*, 625–635. https://doi.org/10.1056/NEJMoa1710504.
14. Gragoudas, E.S.; Adamis, A.P.; Cunningham, E.T.; Feinsod, M.; Guyer, D.R. Pegaptanib for Neovascular Age-Related Macular Degeneration. *N. Engl. J. Med.* **2004**, *351*, 2805–2816. https://doi.org/10.1056/NEJMoa042760.
15. Deev, R.V.; Bozo, I.Y.; Mzhavanadze, N.D.; Voronov, D.A.; Gavrilenko, A.V.; Chervyakov, Y.V.; Staroverov, I.N.; Kalinin, R.E.; Shvalb, P.G.; Isaev, A.A. pCMV-vegf165 Intramuscular Gene Transfer is an Effective Method of Treatment for Patients With Chronic Lower Limb Ischemia. *J. Cardiovasc. Pharmacol. Ther.* **2015**, *20*, 473–482. https://doi.org/10.1177/1074248415574336.
16. Andtbacka, R.H.; Kaufman, H.L.; Collichio, F.; Amatruda, T.; Senzer, N.; Chesney, J.; Delman, K.A.; Spitler, L.E.; Puzanov, I.; Agarwala, S.S.; et al. Talimogene Laherparepvec Improves Durable Response Rate in Patients With Advanced Melanoma. *J. Clin. Oncol.* **2015**, *33*, 2780–2788. https://doi.org/10.1200/jco.2014.58.3377.

17. Adams, D.; Gonzalez-Duarte, A.; O'Riordan, W.D.; Yang, C.-C.; Ueda, M.; Kristen, A.V.; Tournev, I.; Schmidt, H.H.; Coelho, T.; Berk, J.L.; et al. Patisiran, an RNAi Therapeutic, for Hereditary Transthyretin Amyloidosis. *N. Engl. J. Med.* **2018**, *379*, 11–21. https://doi.org/10.1056/NEJMoa1716153.
18. Balwani, M.; Sardh, E.; Ventura, P.; Peiró, P.A.; Rees, D.C.; Stölzel, U.; Bissell, D.M.; Bonkovsky, H.L.; Windyga, J.; Anderson, K.E.; et al. Phase 3 Trial of RNAi Therapeutic Givosiran for Acute Intermittent Porphyria. *N. Engl. J. Med.* **2020**, *382*, 2289–2301. https://doi.org/10.1056/NEJMoa1913147.
19. Garrelfs, S.F.; Frishberg, Y.; Hulton, S.A.; Koren, M.J.; O'Riordan, W.D.; Cochat, P.; Deschênes, G.; Shasha-Lavsky, H.; Saland, J.M.; van't Hoff, W.G.; et al. Lumasiran, an RNAi Therapeutic for Primary Hyperoxaluria Type 1. *N. Engl. J. Med.* **2021**, *384*, 1216–1226. https://doi.org/10.1056/NEJMoa2021712.
20. Ray, K.K.; Wright, R.S.; Kallend, D.; Koenig, W.; Leiter, L.A.; Raal, F.J.; Bisch, J.A.; Richardson, T.; Jaros, M.; Wijngaard, P.L.J.; et al. Two Phase 3 Trials of Inclisiran in Patients with Elevated LDL Cholesterol. *N. Engl. J. Med.* **2020**, *382*, 1507–1519. https://doi.org/10.1056/NEJMoa1912387.
21. Boorjian, S.A.; Alemozaffar, M.; Konety, B.R.; Shore, N.D.; Gomella, L.G.; Kamat, A.M.; Bivalacqua, T.J.; Montgomery, J.S.; Lerner, S.P.; Busby, J.E.; et al. Intravesical nadofaragene firadenovec gene therapy for BCG-unresponsive non-muscle-invasive bladder cancer: A single-arm, open-label, repeat-dose clinical trial. *Lancet Oncol.* **2021**, *22*, 107–117. https://doi.org/10.1016/s1470-2045(20)30540-4.
22. Zhang, S.; Xiao, S.; Sun, Y.; Liu, C.; Su, X.; Li, D.; Xu, G.; Zhu, G.; Xu, B. Clinical Trial of Recombinant Adenovirus-p53 (Gendicine) Combined with Radiotherapy in Nasopharyngeal Carcinoma Patients. *Mol. Ther.* **2006**, *13*, S280. https://doi.org/10.1016/j.ymthe.2006.08.806.
23. Liang, M. Oncorine, the World First Oncolytic Virus Medicine and its Update in China. *Curr. Cancer Drug Targets* **2018**, *18*, 171–176. https://doi.org/10.2174/1568009618666171129221503.
24. Pipe, S.W.; Recht, M.; Key, N.S.; Leebeek, F.W.G.; Castaman, G.; Lattimore, S.U.; Van Der Valk, P.; Peerlinck, K.; Coppens, M.; O'Connell, N.; et al. First Data from the Phase 3 HOPE-B Gene Therapy Trial: Efficacy and Safety of Etranacogene Dezaparvovec (AAV5-Padua hFIX variant; AMT-061) in Adults with Severe or Moderate-Severe Hemophilia B Treated Irrespective of Pre-Existing Anti-Capsid Neutralizing Antibodies. *Blood* 2020, *136*, LBA-6. https://doi.org/10.1182/blood-2020-143560.
25. Russell, S.; Bennett, J.; Wellman, J.A.; Chung, D.C.; Yu, Z.-F.; Tillman, A.; Wittes, J.; Pappas, J.; Elci, O.; McCague, S.; et al. Efficacy and safety of voretigene neparvovec (AAV2-hRPE65v2) in patients with *RPE65* mediated inherited retinal dystrophy: A randomised, controlled, open-label, phase 3 trial. *Lancet* **2017**, *390*, 849–860. https://doi.org/10.1016/S0140-6736(17)31868-8.
26. Day, J.W.; Finkel, R.S.; Chiriboga, C.A.; Connolly, A.M.; Crawford, T.O.; Darras, B.T.; Iannaccone, S.T.; Kuntz, N.L.; Peña, L.D.M.; Shieh, P.B.; et al. Onasemnogene abeparvovec gene therapy for symptomatic infantile-onset spinal muscular atrophy in patients with two copies of *SMN2* (STR1VE): An open-label, single-arm, multicentre, phase 3 trial. *Lancet Neurol.* **2021**, *20*, 284–293. https://doi.org/10.1016/S1474-4422(21)00001-6.
27. Mendell, J.R.; Shieh, P.B.; McDonald, C.M.; Sahenk, Z.; Lehman, K.J.; Lowes, L.P.; Reash, N.F.; Iammarino, M.A.; Alfano, L.N.; Sabo, B.; et al. Expression of SRP-9001 dystrophin and stabilization of motor function up to 2 years post-treatment with delandistrogene moxeparvovec gene therapy in individuals with Duchenne muscular dystrophy. *Front. Cell Dev. Biol.* **2023**, *11*, 1167762. https://doi.org/10.3389/fcell.2023.1167762.
28. Chawla, S.P.; Chua, V.S.; Fernandez, L.; Quon, D.; Blackwelder, W.C.; Gordon, E.M.; Hall, F.L. Advanced Phase I/II Studies of Targeted Gene Delivery In Vivo: Intravenous Rexin-G for Gemcitabine-resistant Metastatic Pancreatic Cancer. *Mol. Ther.* **2010**, *18*, 435–441. https://doi.org/10.1038/mt.2009.228.
29. Aiuti, A.; Roncarolo, M.G.; Naldini, L. Gene therapy for ADA-SCID, the first marketing approval of an ex vivo gene therapy in Europe: Paving the road for the next generation of advanced therapy medicinal products. *EMBO Mol. Med.* **2017**, *9*, 737–740. https://doi.org/10.15252/emmm.201707573.
30. Biffi, A.; Montini, E.; Lorioli, L.; Cesani, M.; Fumagalli, F.; Plati, T.; Baldoli, C.; Martino, S.; Calabria, A.; Canale, S.; et al. Lentiviral hematopoietic stem cell gene therapy benefits metachromatic leukodystrophy. *Science* **2013**, *341*, 1233158. https://doi.org/10.1126/science.1233158.
31. Fumagalli, F.; Calbi, V.; Natali Sora, M.G.; Sessa, M.; Baldoli, C.; Rancoita, P.M.V.; Ciotti, F.; Sarzana, M.; Fraschini, M.; Zambon, A.A.; et al. Lentiviral haematopoietic stem-cell gene therapy for early-onset metachromatic leukodystrophy: Long-term results from a non-randomised, open-label, phase 1/2 trial and expanded access. *Lancet* **2022**, *399*, 372–383. https://doi.org/10.1016/S0140-6736(21)02017-1.
32. Eichler, F.; Duncan, C.; Musolino, P.L.; Orchard, P.J.; De Oliveira, S.; Thrasher, A.J.; Armant, M.; Dansereau, C.; Lund, T.C.; Miller, W.P.; et al. Hematopoietic Stem-Cell Gene Therapy for Cerebral Adrenoleukodystrophy. *N. Engl. J. Med.* **2017**, *377*, 1630–1638. https://doi.org/10.1056/NEJMoa1700554.

33. Thompson, A.A.; Walters, M.C.; Kwiatkowski, J.; Rasko, J.E.J.; Ribeil, J.-A.; Hongeng, S.; Magrin, E.; Schiller, G.J.; Payen, E.; Semeraro, M.; et al. Gene Therapy in Patients with Transfusion-Dependent β-Thalassemia. *N. Engl. J. Med.* **2018**, *378*, 1479–1493. https://doi.org/10.1056/NEJMoa1705342.
